# Oleate activates PLD2 lipase and GEF activity by modulating membrane microdomain dynamics via S-acylation

**DOI:** 10.1016/j.jlr.2025.100939

**Published:** 2025-11-10

**Authors:** Zhiqiang Guo, Karl-Frédérik Bergeron, Catherine Mounier

**Affiliations:** Biological Sciences Department, University of Quebec in Montréal (UQAM), Montréal, Quebec, Canada

**Keywords:** oleate, PLD2, S-acylation, Cdc42, lipid raft, guanine nucleotide exchange factor

## Abstract

Phospholipase D2 (PLD2) plays critical roles in cellular signaling, membrane dynamics, and cancer progression. Oleate (OA) has been shown to activate PLD2 and promote triple-negative breast cancer (TNBC) cell migration, but the underlying molecular mechanisms remain poorly understood. Using confocal microscopy, lipid raft isolation, and S-acylation assays, we show that OA enhanced PLD2 S-acylation at Cys223 and Cys224, disrupting its lipid raft localization, and consequently increasing its colocalization with PIP_2_-enriched microdomains. Furthermore, we identified PLD2 as a guanine nucleotide exchange factor (GEF) for Cdc42, with its GEF activity regulated by OA-dependent S-acylation and lipid raft dynamics. Mutation of the S-acylation sites or disruption of lipid rafts abolished PLD2-mediated Cdc42 activation and filopodia-like cell protrusion formation. These findings reveal a novel regulatory mechanism by which OA modulates PLD2 activity through S-acylation and membrane microdomain reorganization, providing new insights into the regulation of PLD2 in cell migration and signaling.

## Graphical Abstract

**Figure:**
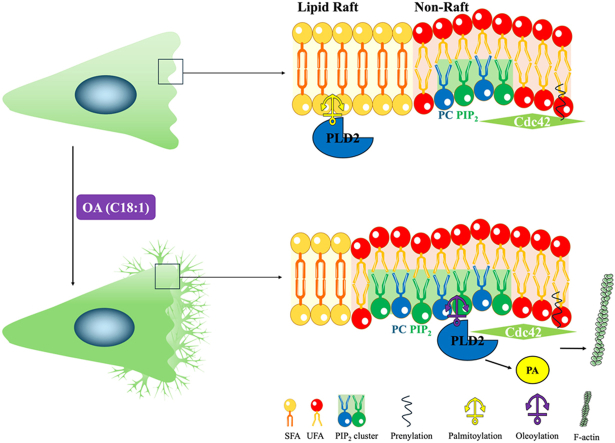

Phospholipase D2 (PLD2) is a key enzyme in lipid signaling, catalyzing the hydrolysis of phosphatidylcholine (PC) to produce phosphatidic acid (PA), a lipid second messenger that plays essential roles in cell proliferation, survival, and migration (1, 2). PLD2 is linked with cancer progression, particularly in metastatic cancers such as triple-negative breast cancer (TNBC), where it enhances cell motility and invasion (3, 4). Our previous studies have highlighted that PLD2 expression is associated with poor clinical outcomes among TNBC patients, and that PLD activity is involved in OA-induced TNBC cell migration and invasion through modulation of cytoskeletal dynamics and cell membrane remodeling (5, 6). Unlike PLD1, which is predominantly found in intracellular compartments, PLD2 localizes mainly at the plasma membrane and exhibits constitutive catalytic activity (7, 8). Of note, PLD2 is inhibited when sequestered in lipid rafts and raft dissociation liberates PLD2, enabling its PC-hydrolyzing activities (9). Additionally, PLD2 is subject to multiple regulatory mechanisms, including phosphorylation, protein-protein interactions, and lipid modifications (10, 11). One of its most intriguing functions beyond its enzymatic role is its guanine nucleotide exchange factor (GEF) activity, specifically activating small GTPases such as Rac2 and RhoA (12, 13). This unique dual functionality positions PLD2 as a key player in cytoskeletal reorganization and cancer metastasis (1, 10).

Oleate (OA), a monounsaturated fatty acid (MUFA), is an important regulator of cancer cell migration and progression (14). It is primarily synthesized by stearoyl-CoA desaturase-1 (SCD1), an enzyme that converts saturated fatty acids (SFAs) into MUFAs. SCD1 is frequently upregulated in various cancers, including breast cancer, and has been linked to increased cell survival, chemoresistance, and metastatic potential (14, 15). OA exerts diverse effects on cancer cells, promoting membrane fluidity, modulating signaling pathways, and influencing lipid metabolism (6). Previous findings have indicated that OA directly activates PLD2, but the underlying molecular mechanisms remain largely unknown (6, 16, 17).

Protein lipidation is a post-translational modification that usually refers to various lipids or lipid-like groups covalently attached to proteins (18, 19). S-acylation is a reversible post-translational modification that involves the attachment of fatty acids (FAs), such as palmitate (C16:0), palmitoleate (C16:1), stearate (C18:0), and OA (C18:1), to cysteine residues via thioester bonds (20, 21, 22, 23). Traditionally referred to as palmitoylation, this process is now recognized as a more diverse lipidation mechanism involving various FAs that differentially regulate protein function, localization, and interactions (20, 21, 22, 23, 24). Unlike other lipid modifications, S-acylation is dynamic, allowing proteins to cycle between membrane-bound and cytosolic states (18). Different FAs confer distinct functional properties to proteins; for instance, palmitate tends to anchor proteins in lipid rafts, whereas OA and palmitoleate promote protein redistribution to non-raft domains (22, 25). This suggests a complex regulatory network in which specific lipid modifications dictate protein behavior within cellular membranes. Beyond S-acylation, cysteine residues can undergo prenylation, another critical lipid modification. This modification entails the attachment of isoprenoid groups, such as farnesyl (C15) or geranylgeranyl (C20) moieties, to cysteine residues near the C-terminus of proteins. Prenylation is critical for the membrane association and protein-protein interactions of various signaling proteins, including Rho family GTPase cell division control protein 42 homolog (Cdc42), which is geranylgeranylated (26, 27, 28).

Membrane microdomains, including lipid rafts and phosphatidylinositol bisphosphate (PIP_2)_ clusters (also referred to as PIP_2_ microdomains or PIP_2_ rafts), are specialized regions of the plasma membrane that organize signaling molecules and regulate cellular functions (29). Lipid rafts are cholesterol- and sphingolipid-rich domains that serve as platforms for receptor clustering and intracellular signaling (30). Proteins localized within lipid rafts often undergo lipid modifications, such as S-acylation, which influence their association with these domains (31). PIP_2_ clusters contain high levels of PI_(4,5)_P_2_, a phospholipid that plays a critical role in actin remodeling and membrane trafficking (32, 33). The lateral distribution of proteins into lipid rafts and PIP_2_ clusters is strongly influenced by membrane lipid composition (34, 35). For example, SFAs within phospholipids promote tighter acyl-chain packing and membrane rigidity, thereby stabilizing cholesterol-rich lipid rafts and facilitating PIP_2_ clustering (36). In contrast, unsaturated FAs—bearing cis-double bonds—present kinks in their acyl chains that disrupt close packing and order, increasing membrane fluidity and disfavoring raft-like ordered domains (9, 36, 37).

Although previous studies have reported that OA activates PLD2, the precise mechanisms remain unclear (16, 17, 38, 39). Like double S-acylated PLD1, human PLD2 is predicted to be S-acylated at Cys223 and Cys224, but the experimental validation of these sites and their functional significance has been lacking. Given the role of S-acylation in protein localization, it is essential to determine whether PLD2 activation involves S-acylation-mediated shuttling between membrane microdomains. Additionally, the functional implications of PLD2’s GEF activity remain underexplored.

In this study, we demonstrate that OA induces PLD2 dissociation from lipid rafts and promotes its translocation to PIP_2_ clusters, a process dependent on S-acylated Cys223 and Cys224. Furthermore, we identify PLD2 as a GEF for Cdc42, with its GEF activity regulated by OA-dependent acylation and lipid raft dynamics. These findings provide a new regulatory mechanism for understanding how OA modulates PLD2 activity, highlighting potential therapeutic strategies for SCD1-and PLD2-dependent TNBC and other metastatic cancers.

## Materials and methods

### Cell lines and culture

MDA-MB-231 cells were a generous gift from Dr Jean-Jacques Lebrun (McGill University, Canada), and HEK293T cells were provided by Dr Benoît Barbeau (UQAM, Canada). Both cell lines were maintained at 37°C in a 5% CO_2_ incubator. MDA-MB-231 cells were cultured in Eagle’s Minimum Essential Medium (EMEM, Wisent #320-005-CL), while HEK293T cells were cultured in Dulbecco’s Modified Eagle Medium (DMEM, Wisent #319-005-CL/#319-010-CL), both supplemented with 10% heat-inactivated fetal bovine serum (FBS, Gibco), 500 U/ml penicillin, and 500 μg/ml streptomycin (Gibco #15070063). Cells between passages 5 and 25 were used. Adherent cells were detached using 0.25% Trypsin-EDTA (Gibco #25200056).

### Reagents and antibodies

Polyethylenimine (PEI) reagents were a kind gift from Dr Nicola Pilon (UQAM). Methyl-β-cyclodextrin (MβCD, AC377110050) and Lipofectamine 3000 (L3000015) were purchased from Invitrogen Thermo Fisher Scientific. Additional reagents and kits were obtained as follows: Cdc42 Activation Assay Kit (#8819, Cell Signaling Technology), Bradford protein assay (Bio-Rad, #5000006), Phalloidin–TRITC (Sigma, #P1951), tris(2-carboxyethyl)phosphine hydrochloride (TCEP; Sigma, #C4706), methoxypolyethylene glycol maleimide (mPEG–Mal; Sigma, #63187), N-ethylmaleimide (NEM; Sigma, #E3876), protease and phosphatase inhibitors (Sigma, #P8340; #P0044), ELOVL6 inhibitor ELOVL6-IN-4 (MedChemExpress, #HY-152947), and SCD1 inhibitor A939572 (Biofine, #37062).

The primary and secondary antibodies used in this study were obtained from the indicated suppliers. The following primary antibodies were used: mouse monoclonal anti-PLD2 (7E4D9, #MA5-31854, Invitrogen), rabbit monoclonal anti-PLD2 (E1Y9G, #13904, Cell Signaling Technology), rabbit polyclonal anti–Caveolin-1 (#PA5-32297, Invitrogen), mouse monoclonal anti– PIP_2_ (2C11, #ab11039, Abcam), rabbit monoclonal anti-GFP (GF28R, #MA5-15256, Invitrogen), and rabbit monoclonal anti-Cdc42 (#8747, Cell Signaling Technology). The secondary antibodies included Alexa Fluor 488 and 647–conjugated antibodies (#4410 and #4412, Cell Signaling Technology) as well as HRP–conjugated anti-mouse and anti-rabbit IgG antibodies (#7076 and #7074, Cell Signaling Technology).

### Cell transfection and treatments

MDA-MB-231 cells were transfected using Lipofectamine 3000, while HEK293T cells were transfected with PEI (1 μg/ml, PEI/DNA ratio 3:1). BSA-conjugated FAs were prepared at a 2:1 M ratio (3.33 mM FA: 1.7 mM BSA) and 100 μM was used for treatment, with FA-free BSA as a control. Inhibitors (A939572, 1 μM; ELOVL6-IN-4, 1 μM) and MβCD (100 μM) were prepared in serum-free medium. And cells were preincubated with these inhibitor cells for 3 h and following FA treatment with the continued presence of them.

### Plasmid construction and sequence analysis

The PA biosensors (GFP-PASS and RFP-PASS plasmids) were kind gifts from Dr David N. Brindley (University of Alberta, Canada), which were developed by Dr Guanwei Du (University of Texas Health Science Center at Houston) based on Spo20-phosphatidic acid–binding domain (PABD) with the addition of a nuclear export sequence to increase sensitivity (40). Human PLD2 cDNA was prepared from HepG2 cells and cloned into the EGFP-C1 vector to generate the EGFP-PLD2 plasmid. A reference plasmid with the same structure, kindly gifted by Dr Min Do Sik (Yonsei University) was also used. Site-directed mutagenesis (41) generated PLD2 mutants (C223A, C224A, C223AC224A, K758R, and ΔCRIB). All constructs were sequence-verified. DNA sequence analysis was performed using ApE (A Plasmid Editor) software (v3.0.6) for sequence annotation, restriction mapping, and primer design. Protein sequence alignments were generated using Clustal Omega (EMBL-EBI) with default parameters. Homology percentages were calculated from pairwise alignments of conserved domains. Primers are shown in Table 1.

**Table 1:** Human PLD2 plasmid construction primers

Primer Name	Sequence
	PLDGACGGCGACCCCTGAGAG
PLD2-WT-R	TCCCCGCGGCTATGTCCACACTTCTAGGGGGATC
PLD2-C223A-F	CCTCACCGCCTGTGGCCGAGACCAAGTTTG
PLD2-C223A-R	CCACAGGCGGTGAGGCCAGGAACACG
PLD2-C224A-F	CACCTGCGCCGGCCGAGACCAAGTTTGTTATCG
PLD2-C224A-R	CGGCCGGCGCAGGTGAGGCCAGGAAC
PLD2-C223AC224A-F	TCACCGCCGCCGGCCGAGACCAAGTTTGTTATCG
PLD2-C223AC224A-R	GGCCGGCGGCGGTGAGGCCAGGAACACG
PLD2-K758R-F	CCACAGCAGGGTGCTCATCGCAGATGACCG
PLD2-K758R-R	AGCACCCTGCTGTGGATGTAGATGAGCTCCG
PLD2-ΔCRIB-F	TCTTTGACGAGGTGCAAGTGGGGAAAAGG
PLD2-ΔCRIB-R	GCACCTCGTCAAAGAGCTGAACAAATGAGATG

### Immunofluorescence and F-actin staining

Cells were seeded at 50%–70% confluency on sterilized coverslips in 6-well plates. After 24 h incubation and subsequent treatments, cells were fixed with 4% paraformaldehyde (PFA) for 15 min. For PIP_2_ staining, fixation included 3% PFA + 0.1% glutaraldehyde followed by 0.1% NaBH_4_ quenching. Cells were permeabilized with 0.1% Triton X-100 for 10 min and blocked with 3% BSA for 1 h. F-actin and nuclei were stained with 50 μg/ml Phalloidin-TRITC and 1 μg/ml DAPI, respectively. Immunostaining followed standard protocols, with overnight incubation at 4°C in primary antibodies as indicated in the Results Section (anti-PLD2 (7E4D9), 1:250; anti-Caveolin-1, 1:500; anti-PIP_2_, 1: 250) and 1 h incubation with Alexa Fluor 488/647 secondary antibodies (1: 500) in the dark. Coverslips were mounted in 90% glycerol/PBS and sealed with clear nail polish.

### Confocal microscopy and image analysis

Images were generated using a Nikon A1 Plus inverted confocal microscope (63× oil objective). Image processing was performed in ImageJ Fiji. PASS fluorescence intensity was analyzed using the line-intensity histogram function, as modified from Lu *et* al. (42). Colocalization analysis was performed using the *Coloc2* plugin, with Pearson's coefficient as a colocalization index. Membrane distribution analysis of PLD2, Caveolin-1, and PIP_2_ were performed by manually tracing the plasma membrane using ImageJ's freehand selection tool, with regions of interest saved in the ROI Manager. Fluorescence intensity measurements were used to calculate mean and standard deviation values, from which the coefficient of variation (CV = standard deviation/mean) was derived to quantify signal heterogeneity. Elevated CV values indicated scattered or clustered distributions, while lower CV values corresponded to homogeneous membrane localization patterns (43).

### Lipid raft isolation

Lipid rafts were isolated using a detergent-free sodium carbonate method adapted from (44). Cells were lysed in 200 mM Na_2_CO_3_ (pH 11 in MBS buffer), sonicated (3 × 10 s, 1500 Hz on ice), and centrifuged (17,000*g*, 10 min). Lysates were mixed with 90% sucrose and overlaid with 35% and 5% sucrose layers, followed by ultracentrifugation (200,000*g*, 20 h, 4 °C, SW41Ti rotor, Beckman Instruments). One mL fractions were collected from top to bottom, and aliquots from each fraction were prepared for Western blot analysis of caveolin-1 and PLD2.

### Acyl-PEG exchange assay

S-acylation of PLD2 was assessed using a modified Acyl-PEG exchange assay based on (45). Briefly, HEK293T cells were transfected with EGFP-PLD2 and incubated in low-glucose DMEM to enhance global protein fatty acid acylation (46). Cells were then lysed in lysis buffer containing acyl thioesterase inhibitor palmostatin B (Palm B, 10 μM) to preserve S-acylation. Proteins were reduced with TCEP, followed by thiol blocking with 25 mM NEM. Hydroxylamine (1 M, pH 7.4) was used to expose nascent thiols. Newly exposed thiols were labeled with 1 mM 5 kDa mPEG-Mal. Samples were analyzed by SDS-PAGE and GFP antibody Western blot to detect mass shift.

### Active Cdc42 pull-down assay

Cdc42 activation was measured using the Cdc42 Activation Assay Kit (Cell Signaling #8819) according to the manufacturer’s instructions. Briefly, cells were lysed, and GTP-bound Cdc42 was affinity-precipitated using a PAK1 PBD-conjugated agarose bead slurry. Beads were washed and bound proteins were eluted in SDS buffer. Samples were resolved by SDS-PAGE, and active Cdc42 (GTP-bound) was detected by Western blot using a Cdc42-specific antibody included in the kit. Total Cdc42 levels were assessed from whole-cell lysates for normalization.

### Western blot

Cells were lysed in RIPA buffer containing protease and phosphatase inhibitors (10 μM Palm B was added for PLD2 S-acylation analysis). After centrifugation (17,000*g*, 10 min), proteins were recovered in the supernatant. Protein concentration was determined by the Bradford protein assay. Proteins were separated on SDS-PAGE and transferred to PVDF membrane, followed by incubation with primary antibodies overnight at 4°C and HRP-conjugated secondary antibodies at room temperature for 2 h. The following primary and secondary antibodies were used: PLD2 (1:500; Cell Signaling Technology, EY19G, #13904), Caveolin-1 (1:1000; #PA5-32297, Invitrogen,), GFP (1:1000; #2555, Cell Signaling Technology), Cdc42 (1:167; #8747, Cell Signaling), HRP-conjugated anti-rabbit IgG (1:2000; Cell Signaling, #7074), and HRP-conjugated anti-mouse IgG (1:2000; #7076, Cell Signaling). Signals were revealed using the ECL substrate (#WBKLS0100, Millipore) and imaged with a digital chemiluminescence detection system (FUSION FX, VILBER). Bands were quantified by the ImageJ Fiji gel analyze tool.

### Statistical analyses

Statistical analyses were carried out using GraphPad Prism version 9.0. The significance of differences between groups was tested using unpaired two-tailed Student's t-tests for pairwise comparisons and one-way ANOVA followed by Dunnett’s multiple comparisons test for datasets with more than two groups. Differences were considered significant when *P* values were < 0.05.

## Results

### OA is a potent activator of PLD in HEK293T and MDA-MB-231 cells

Our previous findings demonstrated that PLD, most likely PLD2, is involved in OA-induced TNBC cell migration and invasion (5, 6). To investigate the effect of different FAs on PLD activation, we utilized PA sensors (RFP/GFP-PASS) to monitor PLD lipase-dependent PA recruitment to the plasma membrane (42). This method offers a highly visual approach to assess PLD activation without the addition of exogenous lipids as substrates (such as labeled PC or phospholipids), which could confound results (47). We used two cell lines as models: HEK293T cells, which have low basal PLD2 expression but high transfection efficiency, served as an overexpression model, while MDA-MB-231 cells, which endogenously express high levels of PLD2 but have lower transfection efficiency, were used as a physiologically relevant model.

**Fig. 1:**
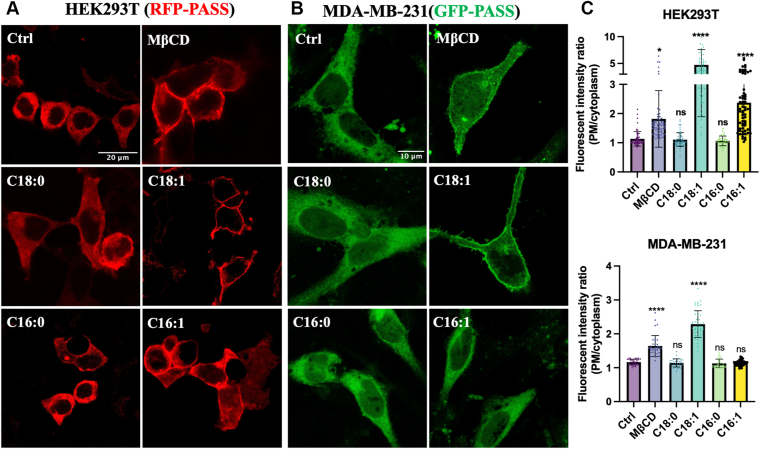
OA induces PLD activation in HEK293T and MDA-MB-231 cells. A, B: Representative confocal microscopy images of the PA sensor (RFP-PASS in HEK293T cells and GFP-PASS in MDA-MB-231 cells) in response to FA treatments. Cells were transiently transfected with RFP/GFP-PASS. After 48 h incubation, cells were starved with serum-free medium for 3 h with 1 μM SCD1 inhibitor A939572 and 1 μM ELOVL6 inhibitor ELOVL6-IN-4 to prevent FA desaturation and elongation (for lipid raft disruption, cells were preincubated with 100 μM MβCD in serum-free medium for 3 h). Cells were then incubated with 100 μM BSA-conjugated FAs (BSA for Ctrl) in the continued presence of these inhibitors. Scale bars in the first micrograph apply to all images in series A or B. C: Quantification of RFP/GFP-PASS fluorescence on the plasma membrane (PM) relative to the cytoplasm. The average (± standard deviation) ratio was calculated from three independent experiments (HEK293T, n ≥ 60; MDA-MB-231, n ≥ 33). Statistical significance was assessed using one-way ANOVA followed by Dunnett’s multiple comparisons test, with significance thresholds defined as follows: ns, not significant, *P* ≥ 0.05; ∗, *P* < 0.05; ∗∗∗∗, *P* < 0.0001.

First, we examined the effects of 4 FAs linked to SCD1 activity (C16:0, C16:1, C18:0, C18:1). To block endogenous FA desaturation and elongation, cells were pretreated with SCD1 and ELOVL6 inhibitors. Confocal imaging revealed a striking increase in plasma membrane localization of PASS in response to OA (C18:1) treatment in both cell lines (Fig. 1). Treatment with the lipid raft disruptor MβCD also resulted in increased PASS recruitment, confirming the implication of lipid rafts in PLD activation within our assay. Interestingly, palmitoleate (C16:1) induced a moderate activation in HEK293T cells but failed to elicit a similar response in MDA-MB-231 cells. These results establish OA (C18:1) as the predominant activator of PLD in both cell types, while the effect of MβCD highlights the central role of lipid rafts in this activation process.

### OA disrupts PLD2 localization to lipid rafts

**Fig. 2:**
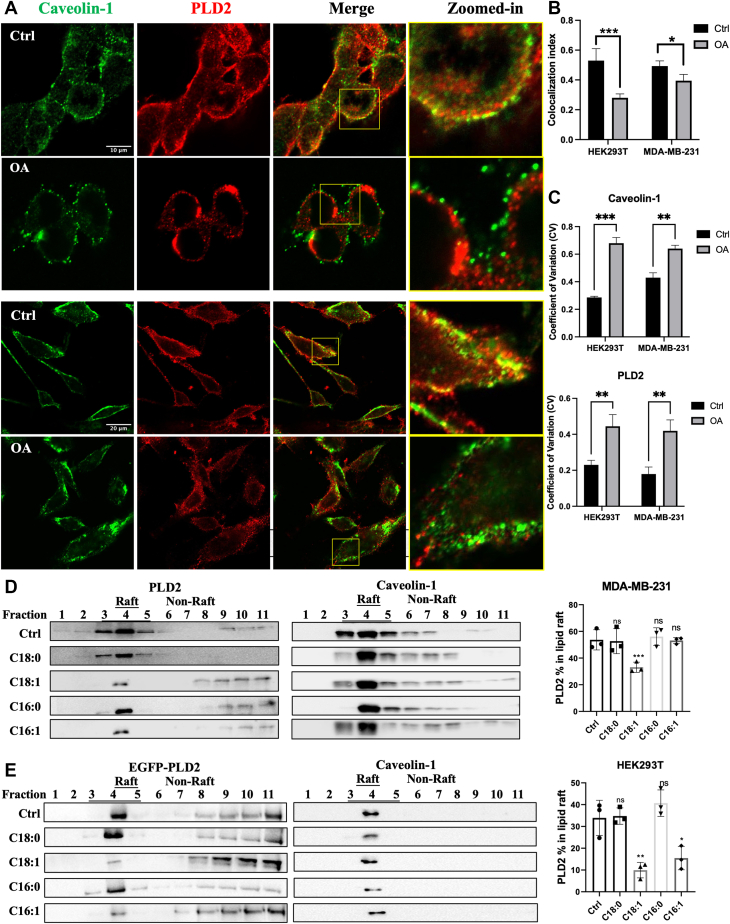
OA disrupts the colocalization of PLD2 with lipid rafts. A: Representative confocal microscopy images showing the colocalization of PLD2 (conjugated with Alexa Fluor 647 secondary antibody, in red) and Caveolin-1 (conjugated with Alexa Fluor 488 secondary antibody, in green) in HEK293T cells and MDA-MB-231 cells treated 3 h with BSA (Ctrl) or 100 μM OA. Scale bars in the first micrograph apply to all images in the series. The zoomed-in channels correspond to the regions highlighted by the yellow rectangles in the merged image. B, C: Quantification of caveolin-1 and PLD2 colocalization (B) and distribution variability (C). Colocalization was assessed using Pearson's correlation coefficient. Distribution heterogeneity was measured by coefficient of variation (CV). D, E: Western blot analysis of lipid raft fractions isolated by sucrose density gradient ultracentrifugation from HEK293T cells expressing EGFP-PLD2 (D) and MDA-MB-231 cells (E). Fractions 3–5 are enriched in lipid rafts, as indicated by the predominant presence of Caveolin-1. Bar charts show the percentage of PLD2 localized in lipid raft fractions under BSA (Ctrl), C18:0, C18:1, C16:0, and C16:1 treatments. Cells were pretreated with 1 μM SCD1 inhibitor A939572 and 1 μM ELOVL6 inhibitor ELOVL6-IN-4 in serum-free DMEM for 3 h to prevent FA desaturation and elongation. 10 μM palmostatin B (Palm B) was used to preserve S-acylated residues in cell lysis buffer. The proportion of PLD2 in raft fractions was calculated as the ratio of the sum of PLD2 in raft fractions (fractions 3–5) to the sum of PLD2 in all 11 fractions. Data are presented as mean ± SEM (n = 3 independent experiments). Statistical significance was assessed using unpaired two-tailed t-tests (B–C) or one-way ANOVA followed by Dunnett’s multiple comparisons test (D–E), with significance thresholds defined as follows: ns, not significant, *P* ≥ 0.05; ∗, *P* < 0.05; ∗∗, *P* < 0.01; ∗∗∗, *P* < 0.001.

Given the findings in the previous section, which demonstrated that OA and lipid rafts are involved in PLD activation, we hypothesized that OA might modulate PLD2 activity by altering its localization within membrane microdomains. To test this, we first examined the colocalization of PLD2 with a lipid raft marker, Caveolin-1, using confocal microscopy. Under control conditions, PLD2 and Caveolin-1 showed strong colocalization at the plasma membrane in both HEK293T and MDA-MB-231 cells (Fig. 2A). However, OA treatment significantly disrupted this colocalization, as evidenced by a more punctate distribution of both proteins (Fig. 2C) and a marked reduction of colocalization (Fig. 2B). These findings demonstrate that OA could disperse the distribution of PLD2 and lipid rafts, and also disrupt their association.

To further validate these observations, lipid raft fractions were isolated using sucrose density gradient ultracentrifugation. Western blot analysis confirmed that PLD2 was predominantly localized in lipid raft-enriched fractions (around fraction 4) under control conditions in both HEK293T and MDA-MB-231 cells (Fig. 2D, E). However, OA treatment significantly reduced the proportion of PLD2 in these raft fractions, consistent with the confocal microscopy results. Notably, among the FAs tested (C18:0, C18:1, C16:0, and C16:1), unsaturated FAs OA (C18:1) and palmitoleate (C16:1) both induced a significant decrease in PLD2 localization within lipid rafts in HEK293T cells, while only OA alone had a pronounced effect in MDA-MB-231 cells (Fig. 2D, E). These results align with the cell-type-specific FA preference of PLD activation data from Figure 1, supporting the hypothesis that OA activates PLD2 by promoting its dissociation from lipid rafts.

### OA enhances recruitment of PLD2 to PIP_2_ clusters

**Fig. 3:**
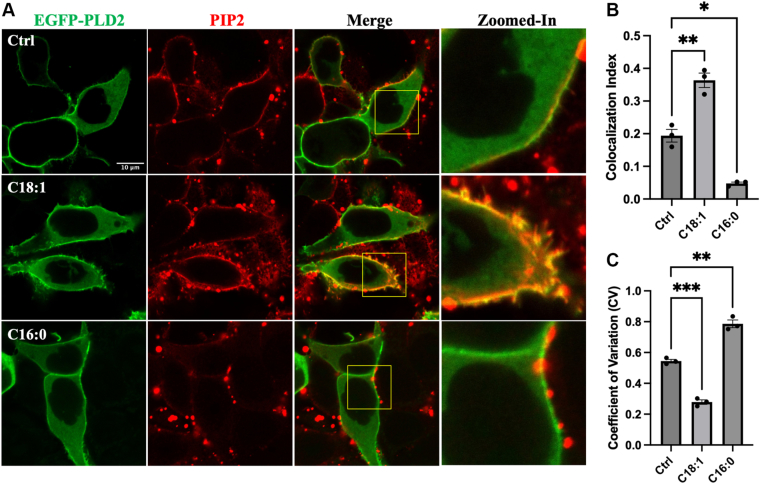
Colocalization analysis of PIP_2_ and PLD2 in HEK293T cells. A: Confocal microscopy images of HEK293T cells expressing EGFP-PLD2. Cells were treated 3 h with BSA (control), 100 μM OA (C18:1), or palmitate (C16:0). Cells were then stained with anti-PIP_2_ antibody (conjugated with Alexa Fluor 647 secondary antibody) to assess colocalization with EGFP-PLD2. The scale bar in the first micrograph applies to all images in the series of panel A. The zoomed-in channels correspond to the regions highlighted by the yellow rectangles in the merged image. B, C: Quantification of PIP_2_ and PLD2 colocalization (B) and PIP_2_’s distribution variability (C). Colocalization index was assessed using Pearson's correlation coefficient. Distribution heterogeneity was measured by coefficient of variation (CV). Data are presented as mean ± SEM (n = 3 independent experiments, n > 20 cells per experiment). Statistical significance was assessed using one-way ANOVA followed by Dunnett’s multiple comparisons test, with significance thresholds defined as follows: ns, not significant, *P* ≥ 0.05; ∗, *P* < 0.05; ∗∗, *P* < 0.01; ∗∗∗, *P* < 0.001.

To further investigate the localization of PLD2 to membrane microdomains, we examined the colocalization of PLD2 with PIP_2_ in HEK293T cells. Due to methodological limitations (both PLD2 and PIP_2_ antibodies are derived from mouse), we utilized an EGFP-PLD2 overexpression system to visualize their colocalization. In untreated cells, PIP_2_ was scattered on the membrane, and PLD2 showed partial colocalization with PIP_2_ (Fig. 3A). Treatment with 100 μM OA (C18:1) for 3 h significantly enhanced a more homogeneous PIP_2_ detection (Fig. 3C) on the membrane and increased its colocalization with PLD2 (Fig. 3B). In contrast, treatment with palmitate (C16:0) sequestered PIP_2_ into fewer, more concentrated puncta on the membrane, reducing the overall PIP_2_ detection area on the membrane and its colocalization with PLD2 (Fig. 3A–C). This differential regulation of PIP_2_ distribution by OA and palmitate aligns with their distinct roles in modulating PLD2 activity (Fig. 1A, C).

### PLD2 is S-acylated at Cys223 and Cys224

**Fig. 4:**
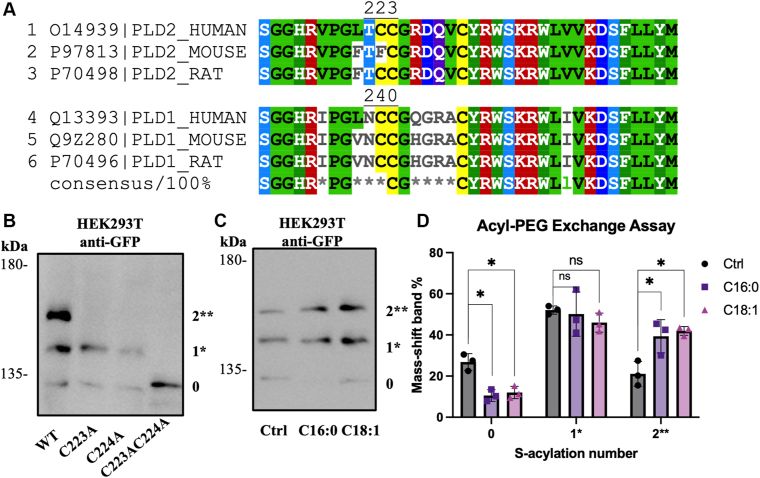
Acyl-PEG exchange assay of PLD2 in HEK293T cells. A: Partial amino acid sequence alignment of canonical PLD1 and PLD2 from *Homo sapiens* (Human), *Mus musculus* (Mouse), and *Rattus norvegicus* (Rat). Numbering indicates positions of predicted or verified S-acylated cysteines (based on SwissPalm database annotations). Conserved residues are colored in the multiple sequence alignment (cysteine residues are highlighted in yellow). B: Western blot analysis of acyl-PEG exchange assay in HEK293T cells transiently transfected with EGFP-PLD2 (WT, C223A, C224A, and C223AC224A). C: Acyl-PEG exchange assay in HEK293T cells expressing EGFP-PLD2-WT treated with BSA (Ctrl), C16:0, or C18:1. Mouse anti-GFP antibody was used to detect EGFP-PLD2. Mass-shift band number were marked with 0, 1∗, 2∗∗. D: Quantitative analysis of mass-shift bands. Band intensities were quantified and expressed as percentage relative to total protein levels (each band/total bands ×100%). Data represent mean ± SEM from three independent experiments. Statistical significance was assessed using one-way ANOVA followed by Dunnett’s multiple comparisons test, with significance thresholds defined as follows: ns, not significant, *P* ≥ 0.05; ∗, *P* < 0.05.

To further investigate the molecular mechanism underlying PLD2 regulation by OA, we explored whether PLD2 undergoes S-acylation, a post-translational modification that could influence its localization and activity. According to the SwissPalm database (48), human PLD2 is predicted to be S-acylated at Cys223 and Cys224 (Fig. 4A), but the experimental validation of these sites is lacking. To address this, we performed an acyl-PEG exchange assay, which replaces acyl groups with a PEG moiety, resulting in a detectable mass shift on western blots (45). We also constructed single Cys mutants (C223A and C224A) and a double Cys mutant (C223AC224 A). Constructs are illustrated in Fig. S1.

We first examined the S-acylation status of wild-type (WT) and mutant human PLD2, expressed in HEK293T cells. To enhance the detection of S-acylation, cells were cultured in low glucose DMEM (46). And a specific inhibitor of acyl-protein thioesterases, palmostatin B (Palm B), was included in the cell lysis buffer to preserve S-acylation (22). As shown in Figure 4B, EGFP-PLD2-WT exhibited two mass-shifts above the original band, indicating double S-acylation. Single mutants (C223A and C224A) resulted in one mass-shift band, while the double mutation (C223AC224A) completely abolished the shifting effects. These findings provide direct experimental evidence that human PLD2 is S-acylated at Cys223 and Cys224.

Next, we investigated whether OA could serve as an acyl donor for PLD2 S-acylation. HEK293T cells expressing WT-EGFP-PLD2 were treated with BSA (Ctrl), palmitate (C16:0), or OA (C18:1) for 3 h before the acyl-PEG exchange assay. Cells were also treated with ELOVL6 and SCD1 inhibitors to prevent modification of supplemented FAs. As shown in Figure 4C, D, both OA and palmitate treatments resulted in stronger mass-shift bands and weaker original bands compared to the control, indicating increased S-acylation of PLD2 following FA treatments. This suggests that OA, like palmitate, can be incorporated into PLD2 via S-acylation.

### S-acylation of PLD2 optimizes membrane association and lipase activity

**Fig. 5:**
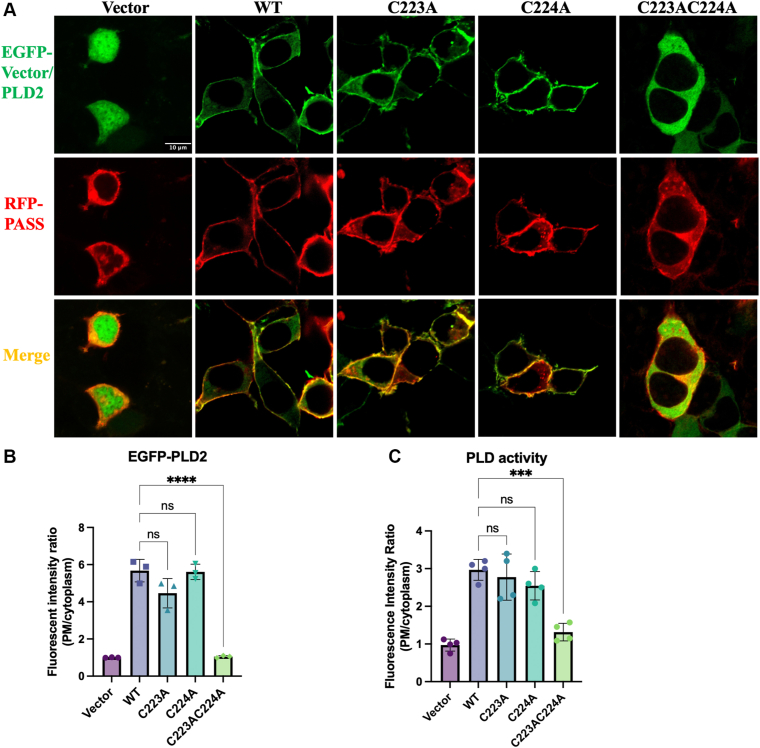
S-acylation sites of PLD2 optimizes membrane localization and lipase activity. A: Confocal microscopy images of HEK293T cells transiently co-transfected with RFP-PASS and EGFP-C1 (vector), EGFP-PLD2 (WT, C223A, C224A, and C223AC224A). The scale bar in the first micrograph apply to all images in the series. B, C: Quantitative analysis of EGFP-Vector/PLD2 (B) and RFP-PASS (C) membrane/cytosolic distribution. Fluorescence intensity ratios (PM/cytoplasm) are presented as mean ± SD from three biological replicates (n > 20 cells per experiment). Statistical significance was assessed using one-way ANOVA followed by Dunnett’s multiple comparisons test to the wild-type controls (WT), with significance thresholds defined as follows: ns, not significant, *P* ≥ 0.05; ∗∗∗, *P* < 0.001; ∗∗∗∗, *P* < 0.0001.

To determine whether the S-acylation of PLD2 at Cys223 and Cys224 influences its lipase activity, we performed a PLD activation assay using a PA sensor (RFP-PASS) in HEK293T cells expressing WT-EGFP-PLD2 or its S-acylation site mutants (C223A, C224A, and C223AC224A). Confocal microscopy imaging revealed that EGFP-PLD2-WT exhibited strong membrane localization in HEK293T cells (Fig. 5A, B). In contrast, the double Cys mutant (C223AC224A) displayed impaired membrane targeting. Single Cys mutants (C223A and C224A) retained membrane association, indicating that S-acylation at either Cys223 or Cys224 is sufficient for PLD2 membrane targeting (Fig. 5A, B). In the PASS channel, which reflects PA production as the measure of PLD lipase activity, expression of all PLD2 constructs (WT and mutants) resulted in higher PA membrane recruitment compared to the vector control (Fig. 5A, C). Therefore, PLD2 retains basal lipase activity even in the absence of S-acylation and membrane localization. However, the double Cys mutant (C223AC224A) exhibited significantly lower activation compared to WT (Fig. 5A, C). These results demonstrate that S-acylation at both Cys223 and Cys224 is essential for optimal PLD2 membrane localization and lipase activity.

### PLD2 lipid raft targeting is regulated by S-acylation

**Fig. 6:**
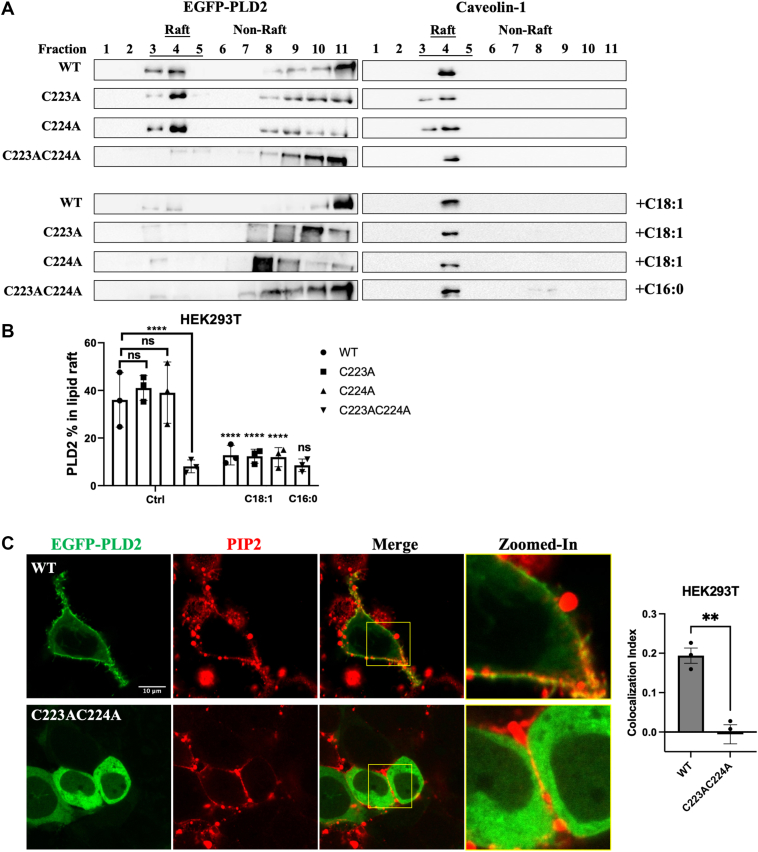
The impact of PLD2 S-acylation site mutation on its targeting to lipid rafts. A: Western blot analysis of lipid raft fractions isolated by sucrose density gradient ultracentrifugation from HEK293T cells expressing EGFP-PLD2 (WT, C223A, C224A, and C223AC224A). Cells were starved with serum-free medium and preincubated with 1 μM SCD1 inhibitor A939572 and 1 μM ELOVL6 inhibitor ELOVL6-IN-4 for 3 h to prevent FA desaturation and elongation. Cells were then treated with BSA (Ctrl groups) or FAs (100 μM OA for WT, C223A, C224A; 100 μM palmitate for C223AC224A) for 3 h. Fractions 3–5 pinpoint lipid raft-enriched fractions, as indicated by the presence of Caveolin-1. B: Quantification of the proportion of PLD2 in raft fractions relative to the total PLD2 across all fractions. C: Confocal microscopy images of HEK293T cells expressing EGFP-PLD2-WT and -C223AC224A. Cells were then stained with anti-PIP_2_ antibody (conjugated with Alexa Fluor 647 secondary antibody) to assess colocalization with EGFP-PLD2. The scale bar in the first micrograph applies to all images in the series of panel. The zoomed-in channels correspond to the regions highlighted by the yellow rectangles in the merged image. Colocalization index was assessed using Pearson's correlation coefficient. Data are presented as mean ± SEM (n = 3 independent experiments). Statistical significances were analyzed using one-way ANOVA followed by Dunnett’s multiple comparisons test to WT in Ctrl group for (B). Unpaired two-tailed Student's t-tests were used in FA treated group to compare the FA treated group and the corresponding BSA-treated control for (B) and WT versus C223AC224A for (C). Significance thresholds were defined as: ns, not significant, *P* ≥ 0.05; ∗∗, *P* < 0.01; ∗∗∗∗, *P* < 0.0001.

To further investigate the role of S-acylation in PLD2 membrane localization, we performed lipid raft isolation in HEK293T cells expressing EGFP-PLD2-WT or its S-acylation site mutants (C223A, C224A, and C223AC224A). Consistent with the findings presented above in Figure 5, WT and single mutant (C223A and C224A) constructs showed significant presence in lipid raft-enriched fractions (fractions 3–5) under basal conditions (Fig. 6A, B). In contrast, the double mutant (C223AC224A) exhibited a marked decrease in lipid raft localization and a corresponding increase in non-raft fractions, underscoring the importance of S-acylation at both Cys223 and Cys224 for PLD2 association with lipid rafts.

To examine whether OA treatment affects the lipid raft localization of PLD2 single mutants, HEK293T cells expressing PLD2-WT and single Cys mutants (C223A and C224A) were treated with 100 μM OA for 3 h. OA treatment significantly reduced the presence of single Cys mutants in lipid raft fractions (Fig. 6A, B), indicating that S-acylation at either Cys223 or Cys224 is sufficient to retain OA-mediated regulation of PLD2 raft localization. Since the double mutant (C223AC224A) has an impaired association with lipid rafts, we tested whether palmitate treatment could restore its colocalization with lipid rafts. However, it failed to rescue the lipid raft association of the double Cys mutant (Fig. 6A, B), confirming that Cys223 and Cys224 are the key sites required for PLD2 localization to lipid rafts.

In addition, we also analyzed the impact of PLD2 S-acylation on its association with PIP_2_ clusters. In HEK293T cells expressing the double S-acylation Cys mutant (C223AC224A), PLD2-C223AC224A has impaired membrane localization, resulting in minimal colocalization with PIP_2_ (Fig. 6C). This further supports the critical role of S-acylation at Cys223 and Cys224 in anchoring PLD2 to the membrane microdomain and facilitating its interaction with PIP_2_.

Taken together, these results demonstrate that S-acylation at Cys223 and Cys224 is essential for PLD2 localization with lipid rafts and PIP_2_ clusters. Collectively, these findings provide strong evidence that S-acylation at Cys223 and Cys224 is a critical determinant of PLD2 function, linking its membrane localization, regulation by FAs, and enzymatic activity.

### PLD2 functions as a GEF for Cdc42 activation

**Fig. 7:**
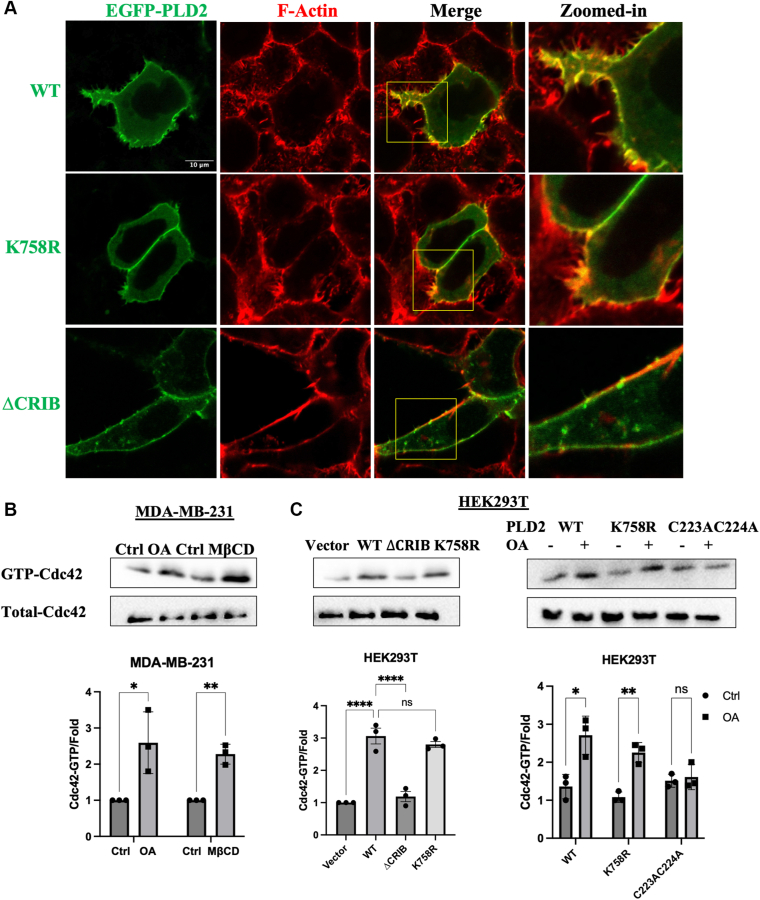
PLD2 functions as a GEF for Cdc42 activation. A: Confocal microscopy images of HEK293T cells overexpressing EGFP-PLD2 (WT, K758R, ΔCRIB). Cells were stained with Phalloidin-TRITC to visualize filopodia-like cell protrusions. The scale bar in the first micrograph applies to all images in the series. The zoomed-in channels correspond to the regions highlighted by the yellow rectangles in the merged image. B, C: Western blot analysis of GTP-Cdc42 (active form) and total Cdc42 (input) in MDA-MB-231 cells (B) and HEK293T cells (C). MDA-MB-231 cells were treated with BSA (Ctrl)/100 μM OA for 10 min or with DMSO (Ctrl)/100 μM MβCD for 3 h HEK293T cells expressing EGFP-vector/EGFP-PLD2 (WT/ΔCRIB/K758R) were lysed to detect basal level of Cdc42 by Western blot. HEK293T cells expressing PLD2-WT/K758R/C223AC224A were treated with BSA (Ctrl)/100 μM OA for 10 min. Data are presented as mean ± SEM (3 independent experiments). Statistical significance was determined by unpaired two-tailed Student's t-tests with their corresponding control groups (B and right panel in C) or one-way ANOVA followed by Dunnett’s multiple comparisons test to WT (left panel in C). Significance thresholds were defined as: ns, not significant, *P* ≥ 0.05; ∗, *P* < 0.05; ∗∗, *P* < 0.01; ∗∗∗∗, *P* < 0.0001.

Previous studies have identified PLD2 as a GEF for Rac2 and RhoA (12, 13), and our earlier work demonstrated that PLD2 is involved in OA-induced Cdc42-dependent filopodia formation (6). To investigate whether PLD2 could also act as a GEF for the Cdc42 GTPase, we first examined the role of PLD2 in Cdc42 activation using filopodia formation assays. In Figure 7A, confocal microscopy images of HEK293T cells expressing EGFP-PLD2(WT, K758R (a lipase-inactive mutant (13), ΔCRIB (deletion of aa 263–266 (13), a GEF inactive mutant); Mutant constructs are shown in Fig. S1) revealed that WT and lipase inactive construct induced significant filopodia-like cell protrusions, while the GEF inactive construct did not. This indicates that the GEF regulation domain, but not the lipase catalytic domain, is critical for PLD2-mediated Cdc42 dependent filopodia-like protrusion formation.

To directly assess Cdc42 activation, we performed a pull-down assay to detect GTP-bound Cdc42 (active form). In MDA-MB-231 cells, treatment with OA or the lipid raft disruptor MβCD increased GTP-Cdc42 levels (Fig. 7B). In HEK293T cells, expression of PLD2-WT and the lipase inactive PLD2-K758R significantly increased GTP-Cdc42 levels, while the GEF inactive ΔCRIB construct showed a much weaker effect, confirming that PLD2 acts as a GEF for Cdc42 and that its lipase activity is dispensable for this function (Fig. 7C). Next, we investigated whether S-acylation is required for PLD2’s GEF activity. OA treatment increased GTP-Cdc42 levels in HEK293T cells expressing PLD2-WT and PLD2-K758R but failed to do so in cells expressing the double S-acylation mutant C223AC224A (Fig. 7C). This demonstrates that PLD2’s GEF activity is also impacted by the proper localization via S-acylation. Taken together, these results establish PLD2 as a GEF for Cdc42 activation and reveal that OA-dependent S-acylation and membrane lipid microdomain localization are critical for regulating PLD2’s GEF function.

## Discussion

This study delineates a novel molecular mechanism by which OA activates PLD2 through S-acylation, regulating its lipase and GEF activities via membrane microdomain translocation dynamics. We demonstrate that OA induces PLD2 dissociation from lipid rafts and promotes its translocation to PIP_2_-enriched microdomains, where it accesses its substrate and exerts enzymatic functions. This process is dependent on S-acylation at Cys223 and Cys224, as mutation of these sites abolishes PLD2 membrane localization and OA-induced activation. Interestingly, while single Cys mutations at either Cys223 or Cys224 retained partial function, the double Cys mutation resulted in a complete loss of activity, indicating a cooperative role of these residues in PLD2 regulation.

Our results show that palmitoleate (C16:1) induces moderate PLD activation and PLD2 raft association in HEK293T cells but failed to elicit a similar response in MDA-MB-231 cells, suggesting that the FA species response of PLD2 activation may depend on cell-type–specific factors (Fig. 1, Fig. 2 and 2). Preference for specific FAs in S-acylation depends on the cellular lipid pool as well as the expression/activity of acyltransferases and thioesterases (25). For example, *Triola* and colleagues used hydroxylamine probes to profile S-acylated fatty acids across various cell types and found that palmitate (C16:0), palmitoleate (C16:1), and OA (C18:1) were the most commonly incorporated FAs in HEK293T and HeLa cells. In contrast, in mouse neuroblastoma N2a cells, stearate (C18:0) was more prominent, while palmitoleate (C16:1) levels were significantly lower. Notably, the incorporation of unsaturated FAs into proteins for S-acylation correlated with SCD1 activity, suggesting a direct link between FA metabolism and lipid modification of proteins (21). The specificity of S-acylation also depends on the substrate preferences of ZDHHC family acyltransferases. Some, like ZDHHC3 and ZDHHC7, show broad substrate specificity, while others, such as ZDHHC9 and ZDHHC17, exhibit highly selective activity (e.g., toward H-Ras and SNAP-25, respectively) (49). While HEK293T cells generally express most ZDHHC isoforms at moderate and balanced levels (50, 51), cancer cells—including breast cancer—often display skewed ZDHHC expression patterns, with certain enzymes upregulated or downregulated. This imbalance can lead to biased S-acylation substrate selection and may impact the functional outcomes of lipid modifications (52, 53, 54, 55). Although data on ZDHHC expression in MDA-MB-231 cells remain limited, studies have implicated ZDHHC3, 5, 15, and 20 in cancer-related S-acylation processes (50, 55, 56, 57). Interestingly, even within the same ZDHHC enzyme, substrate specificity can vary across cell types. For instance, ZDHHC20 was found to be expressed in both HEK293T and MDA-MB-231 cells; However, it modified 231 substrates in HEK293T cells versus only 50 in MDA-MB-231 cells, with just 30 shared between the two cell types (50). Breast cancer cells are also characterized by elevated SCD1 and ELOVL6 activity, which contribute to a higher MUFA pool—especially OA—compared to non-cancerous cells (5, 58, 59). In MDA-MB-231 cells, a recent single-cell lipidomic analysis confirmed that OA is the dominant unsaturated FA, whereas C16:1 is present only at minor levels (60). These differences in lipid metabolism and S-acylation enzyme expression likely explain the cell-line-specific FA responses we observed—the weak palmitoleate response in MDA-MB-231 cells compared to HEK293T. In contrast, OA consistently activates PLD2 in both cell lines, suggesting that OA acts as a widely utilized S-acylation lipid—like palmitate—but with distinct functional consequences. Specifically, OA's ability to disrupt PLD2’s lipid raft association and enhance its colocalization with PIP_2_ clusters appears to underlie its potent regulatory role in cancer cell signaling and migration.

Our findings provide direct evidence that the type of FA modification differentially regulates PLD2 localization and function through distinct membrane microdomains. Specifically, we observed that treatment with OA led to a more homogenous distribution of PIP_2_ across the membrane and significantly enhanced the colocalization of PLD2 with PIP_2_ clusters (Fig. 3). In contrast, palmitate treatment caused PIP_2_ to condense into fewer, larger puncta and reduced PLD2 colocalization. These results suggest that OA promotes the redistribution of PLD2 away from lipid rafts and toward PIP_2_ clusters, where its enzymatic activity is likely optimized. This is consistent with our caveolin-1 imaging data, which showed that OA disrupts PLD2 association with lipid rafts (Fig. 2), while palmitate reinforces it. Importantly, our observations align with previous studies demonstrating that palmitoylation tends to anchor proteins in cholesterol-rich lipid rafts, whereas unsaturated FAs, like OA, destabilize these domains and favor translocation to more dynamic, signaling-active regions such as PIP_2_ clusters (9, 22). The OA-induced dissociation of PLD2 from lipid rafts and enhanced localization to PIP_2_ clusters supports a model in which diversified S-acylation serves as a regulatory switch that redirects PLD2 to membrane microdomains, more conducive to its signaling role. Moreover, the fact that PIP_2_ is typically excluded from rigid lipid rafts but becomes enriched in more fluid, unsaturated regions (61, 62) further reinforces our interpretation that OA promotes a membrane environment favorable for PLD2 activation. Thus, our data provide mechanistic insight into how FA-driven lipid remodeling alters the membrane spatial dynamics of PLD2, with functional consequences for downstream signaling.

OA has been specifically implicated in PLD2 activation (16, 17, 38, 39). PLD1, the other major isoform of PLD, also undergoes S-acylation, but its regulation differs from PLD2. PLD1 is primarily palmitoylated at two cysteine residues (Cys240 and Cys241, Fig. 4A), which facilitate its translocation from the cytosol to the membrane (63, 64). Unlike PLD2, which is constitutively membrane-associated, PLD1 requires palmitoylation for membrane localization and enzymatic activation (7, 65). This difference in regulation may reflect the distinct roles of palmitate in regulating PLD1 and PLD2 via S-acylation. Palmitoylation serves as an activation mechanism for PLD1 while it diminishes PLD2 activity by anchoring it in lipid rafts to isolate it from its substrate. Our findings align with previous studies showing that the type of FA associated with proteins during S-acylation impacts membrane microdomain localization and S-acylated protein functions (22).

Our study provides the first direct experimental evidence that human PLD2 is S-acylated at Cys223 and Cys224, a modification essential for its membrane localization and function (Fig. 3, Fig. 5, Fig. 6, 5 and 6). Furthermore, our data indicate that OA enhances PLD2 S-acylation (Fig. 4), suggesting that OA serves as an acyl donor or indirectly promotes S-acylation. Given that S-acylation is reversible, PLD2 localization and activity may be dynamically regulated in response to metabolic cues. However, due to methodological limitations, the acyl-PEG exchange assay cannot distinguish the specific FA species involved in S-acylation. Future studies using mass spectrometry and lipidomics will be necessary to precisely identify the fatty acyl chains and better elucidate their regulatory roles. Interestingly, our results also show single Cys mutants (C223A or C224A) retain function, while the double Cys mutant (C223AC224A) loses membrane localization and lipid raft association (Fig. 2). This suggests that S-acylation at either Cys223 or Cys224 is sufficient for PLD2 membrane targeting and function, but double S-acylation enhances stability and efficiency. This is consistent with studies on other S-acylated proteins, where multiple S-acylation sites increase membrane affinity and functional robustness. The biochemical basis for membrane binding is clear: two FAs are better than one (66, 67). For example, G_αq_ is doubly palmitoylated, and mutation of any single site reduces its membrane localization and function, while double mutations abolish activity entirely (68). Similarly, mutation of growth-associated protein 43 has shown that double palmitoylation is required for its membrane association while single mutants only impair it (69). In the case of PLD2, the conservation of Cys223 and Cys224 across species further highlights their functional significance. Rat PLD2, like human PLD2, is S-acylated at Cys223 and Cys224, while mouse PLD2, which lacks Cys223, retains only one S-acylation site at Cys224. Despite this difference, mouse PLD2 remains functional, indicating that a single S-acylation site might be sufficient for membrane targeting and activity. This conservation across species indicates that S-acylation is a fundamental regulatory mechanism for PLD2.

Beyond its lipase activity, we identify for the first time PLD2 as a GEF for Cdc42, a key regulator of actin cytoskeleton remodeling and filopodia formation. The lipase activity of PLD2 is dispensable for its GEF function, as the lipase-inactive mutant (K758R) retains the ability to activate Cdc42 and induce filopodia-like protrusion formation. However, the GEF-inactive mutant (ΔCRIB) fails to activate Cdc42, underscoring the importance of the GEF function domain in cytoskeletal rearrangements. The CRIB (Cdc42/Rac Interactive Binding) domain is identified as a highly conserved protein domain that mediates interactions with the small GTPase Cdc42 and, to a lower extent with Rac. (70, 71). The discovery of 2 CRIB domains in PLD2’s PX domain suggests that PLD2 plays a specialized role in Cdc42-dependent processes such as filopodia formation and cell migration, consistent with our previous findings that OA promoted TNBC cell migration by enhancing Cdc42-dependent filopodia formation (72).

As discussed above, the lipase activity of PLD2 is regulated by S-acylation dependent lipid rift association. We wonder if it is also involved in its GEF activity. As is shown in Figure 7, in MDA-MB-231 cells, disrupting lipid rafts using MβCD increased Cdc42 activation. In HEK293T cells, the C223AC224A PLD2 mutant lost OA-induced Cdc42 activation. These results indicate that PLD2’s GEF activity is also regulated by membrane microdomain dynamics via S-acylation. Cdc42, which is prenylated and membrane-anchored, is known to be excluded from lipid rafts (73, 74, 75). S-acylation of PLD2 promotes its dissociation from lipid rafts, potentially facilitating interaction with prenylated Cdc42. Additionally, PA, the lipase product of PLD2, could further regulate GEF activity by recruiting and stabilizing signaling complexes at the membrane (76). Collectively, our results suggest that OA induced S-acylation of PLD2 has a dual regulation effect on both lipase and GEF activity.

Based on our findings, we propose a novel regulatory mechanism in which OA-induced S-acylation of PLD2 promotes its dissociation from lipid rafts and translocation to PIP_2_ clusters (as shown in the graphical abstract). This redistribution enhances PLD2 accessibility to its substrate PC, facilitating PA production. Simultaneously, dissociation from lipid rafts facilitates PLD2 interaction with prenylated Cdc42 at the membrane, promoting GTP exchange and Cdc42 activation. Additionally, PA might also influence Cdc42 activation via crosstalk with PLD2’s GEF activity (76). This provides a mechanistic link between OA-induced PLD2 activation, Cdc42-dependent cytoskeletal rearrangements, and cell migration.

Together, our findings reveal OA as a selective activator of PLD2 through S-acylation and lipid raft remodeling, linking FA metabolism to lipid signaling and cytoskeletal regulation. The distinct and opposing effects of OA (MUFA) and palmitate (SFA) on PLD2 activation highlight a metabolic cue–SCD1-dependent mechanism underlying PLD2 regulation. In the context of TNBC, where OA is abundant in the tumor microenvironment, this regulatory pathway likely contributes to enhanced migratory behavior. By positioning PLD2 S-acylation at the intersection of lipid metabolism and actin remodeling, our study highlights a mechanism that may be exploited therapeutically in TNBC and other cancers with dysregulated lipid signaling. Targeting PLD2 S-acylation represents a promising new strategy, especially considering the limited efficacy of current approaches that mainly focus on blocking PLD lipase activity.

## Data availability

All data supporting the findings of this study are included in the manuscript and its supplementary materials. Additional raw datasets generated and analyzed during the current study are available from the corresponding author upon reasonable request. No software code was developed for this study.

## Supplemental data

This article contains supplemental data.

## Conflict of interest

The authors declare that they do not have any conflicts of interest with the content of this article.
