# Supplementary material for: Oleate activates PLD2 lipase and GEF activity by modulating membrane microdomain dynamics via S-acylation
Source: J Lipid Res. 2025 Nov 10;66(12):100939. doi: 10.1016/j.jlr.2025.100939 (PMC12743522; doi:10.1016/j.jlr.2025.100939)
Supplement: Supplementary Data [file mmc1.docx]

## Supplementary Data:


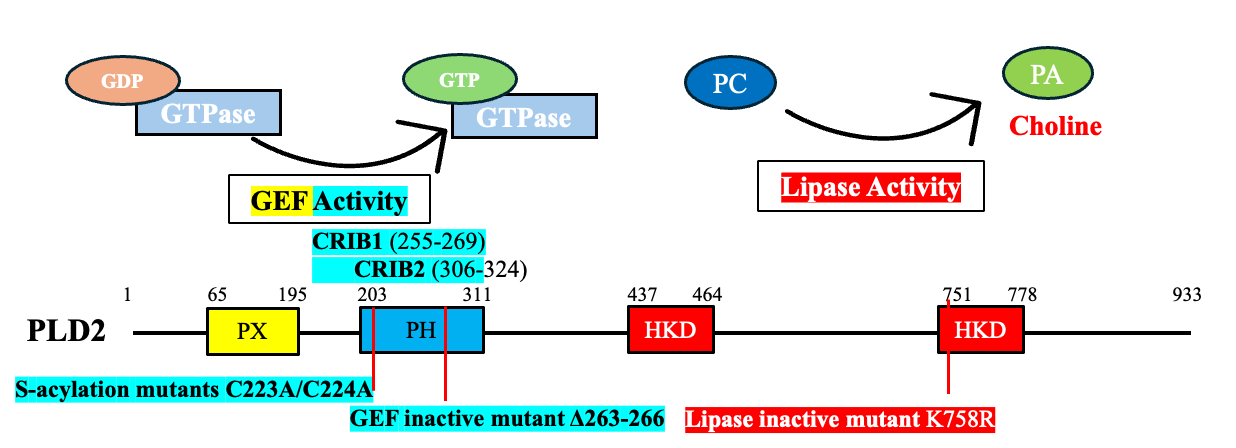


**S1. Schematic representation of the major structural domains and mutants of human PLD2.**

The mutants generated in this study: S-acylation-deficient mutant: PLD2-C223A/C224A, GEF-activity-deficient mutant: PLD2-Δ263–266 (impairs GTPase interaction), catalytically inactive mutant: PLD2-K758R (abolishes lipase activity).
